# The Role of Estrogen Receptor α in Response to Longitudinal Bone Growth in ob/ob Mice

**DOI:** 10.3389/fendo.2021.749449

**Published:** 2021-12-01

**Authors:** Lin-Yu Jin, Chen Guo, Shuai Xu, Hai-Ying Liu, Xin-Feng Li

**Affiliations:** ^1^ Department of Spinal Surgery, Peking University People’s Hospital, Peking University, Beijing, China; ^2^ Department of Spinal Surgery, Renji Hospital, School of Medicine, Shanghai Jiaotong University, Shanghai, China

**Keywords:** estrogen receptor, leptin, skeletal longitudinal growth, growth plate, endochondral bone formation

## Abstract

The absence of leptin results in contrasting growth pattern of appendicular and axial bone growth in ob/ob mice. Endochondral bone formation is an important procedure of growth plate determining the bone growth, where this procedure is also regulated by estrogen and its receptor (ER) signaling pathway. The present study is undertaken to explore the roles of ERs in regulating the different growth patterns in ob/ob mice. In this study, C57BL/6 female mice were used as wild-type (WT) mice; ob/ob mice and WT mice were age-matched fed, and bone length is analyzed by X-ray plain film at the 12 weeks old. We confirm that ob/ob mice have shorter femoral length and longer spine length than WT mice (*p* < 0.05). The contrasting expression patterns of chondrocyte proliferation proteins and hypertrophic marker proteins are also observed from the femur and spinal growth plate of ob/ob mice compared with WT mice (*p* < 0.01). Spearman’s analysis showed that body length (axial and appendicular length) is positively related to the expression level of ERα in growth plate. Three-week-old female ob/ob mice are randomized divided into three groups: 1) ob/ob + ctrl, 2) ob/ob + ERα antagonist (MPP), and 3) ob/ob + ERβ antagonist (PHTPP). Age-matched C57BL/6 mice were also divided into three groups, same as the groups of ob/ob mice. MPP and PHTPP were administered by intraperitoneal injection for 6 weeks. However, the results of X-ray and H&E staining demonstrate that leptin deficiency seems to disturb the regulating effects of ER antagonists on longitudinal bone growth. These findings suggested that region-specific expression of ERα might be associated with contrasting phenotypes of axial and appendicular bone growth in ob/ob mice. However, ER signaling on longitudinal bone growth was blunted by leptin deficiency in ob/ob mice, and the underlying association between ERs and leptin needs to be explored in future work.

## Introduction

Leptin, a protein encoded by obese gene (ob), has been shown to have regulative effects on longitudinal bone growth, spongiosa maturation, and bone turnover between cortical and cancellous bones (1). The mammalian second growth spurt period occurs at puberty. One of the characterizations for the growth spurt is rapid skeletal longitudinal growth. Results in a clinical investigation, in order to clarify the relationship between anthropometric data and concentrations of serum leptin in the catch-up growth procedure, showed that increasing leptin concentrations might trigger catch-up growth in children recovering from malnutrition (2). Furthermore, leptin was considered as an important facilitator in early puberty (3). In addition, findings of leptin deficiency in mice and humans leading to abnormal bone development suggested that leptin plays a novel role in the linear growth of bone (4–7). Endochondral bone formation is the key procedure for growth plate (GP), in which GP chondrocytes undergo a series of organized procedures including proliferation, maturation, hypertrophic differentiation, and calcification (8–10). These complex and delicate programs of endochondral bone formation are responsible for the skeletal elongation. The femoral GP of ob/ob mice is abnormal combined with poorly organized collagen fibril and decreased the expression level of type X collagen (7). Further lab investigations showed that leptin could enhance the proliferation and differentiation of GP chondrocytes *ex vivo* (11). Additional leptin treatment increased condyle height in an organ model (12). Importantly, supplementation of leptin to ob/ob mice could reverse the defects in femoral GP and bone morphology (7, 12, 13). However, interesting different growth patterns have been shown in ob/ob mice with a shorter femora length and a longer vertebral length than wild-type (WT) mice (7), while the underlying mechanism of this contrasting phenotypes still remains unclear.

Although leptin played an important role in the abnormal skeletal bone growth, leptin was a multiple-effect hormone, affecting energy metabolism, appetite, thermogenesis, immune system, and neuroendocrine (14, 15), each of which could independently determine the growth pattern of skeletal bone longitudinal growth (16). Leptin is responsible for the secretion of gonadotropin-releasing hormone, and leptin deficiency directly leads to a reduced level of estrogen in ob/ob mice (17). Estrogen and its receptor (ERα and ERβ) signaling pathway played an essential role in skeletal growth, and the absence of estrogen in rats presented an accelerated bone elongation as compared with that in normal female rats (18, 19). Effects of different ERs (α and β) on the axial and appendicular bone elongation were distinct, which were proved by ER knock-out mice. ERα knock-out mice showed a decreasing bone growth, while mice with inactivation of ERβ had increased appendicular bone growth (20–22). Our previous study also showed that blocking different ERs presented opposite effects on the appendicular longitudinal growth (23). Recently, a crosstalk effect between leptin and ERs in the endochondral bone formation has been reported in our previous study (24), and a stage-specific effect of leptin on the ER expression was obvious during the hypertrophic differentiation stage in a model of chondrocyte differentiation *in vitro* (25). The integration of estrogen and leptin signaling, which is responsible for contrasting growth patterns in appendicular and axial longitudinal growth *via* ERs, is still unknown.

Therefore, we hypothesize that leptin deficiency may influence the ER expression in axial and appendicular GP *in vivo*, contributing to the contrasting phenotypes of skeletal growth in ob/ob mice. The current study was designed to determine the role of ERs in the skeletal longitudinal growth of leptin deficiency mice. We examine the femur and spinal GP using densitometric, histomorphometric, and immunohistochemical techniques to expand our current understanding of how the absence of leptin affects axial and appendicular growth.

## Methods and Materials

### Animals

Three-week-old female B6/JGpt-Lep^em1Cd25/Gpt^ mice on the C57BL/6 background (ob/ob mice, n = 6/group) and 3-week-old female C57BL/6 (n = 6/group) mice were purchased from Nanjing GemPharmatech Co., Ltd. Animals were housed and kept in 12-h daylight and nightlight conditions with standard mouse feed and sterile water. Our animal protocol was approved by the animal experiment ethical committee of Peking University People’s Hospital.

### Experimental Procedures

In total, 24 C57BL/6 mice and 24 ob/ob mice were purchased from GemPharmatech Co., Ltd. (Jiangsu, China) and were randomly divided into eight groups. In the first stage, C57BL/6 was used as WT (n = 6), ob/ob mouse was used as ob/ob (n = 6). These two groups of mice did not receive any drug treatment and were euthanized for tissue collection. In order to determine the role of ERs in skeletal growth of ob/ob mice, the second stage was performed as follows: WT, ob/ob control (ob/ob + ctrl), ob/ob mice treated with methyl-piperidino-pyrazole, an ERα-selective antagonist (ob/ob + MPP), and ob/ob mice were treated with 4-(2-phenyl-5,7-bis(trifluoromethyl)pyrazolo(1,5-*a*)pyrimidin-3-yl)phenol, an ERβ-selective antagonist (ob/ob + PHTPP). After 1 week of adaptive feeding, mice were treated with different kinds of ER antagonists during the puberty of female mice (4 to 10 weeks). The control group ob/ob mice were subcutaneously injected with 100 μl of saline solution. The MPP group mice and PHTPP group mice were intraperitoneally injected with MPP [0.3 mg/kg/day, dissolved in 1‰ dimethyl sulfoxide (DMSO)] and PHTPP (0.3 mg/kg/day, dissolved in 1‰ DMSO), respectively, according to previous studies (23, 26). All the groups received injections at 5 days/week for 6 weeks. The body weight of each mouse was recorded every week.

### X-Ray Radiography Image

At the end of each stage, X-ray radiography (Shanghai United Imaging Healthcare Co., Ltd. Shanghai, China) was used to get the images of all mice, which were in the prone position. Femur length and spine (L1–L6) length were quantified using ImageJ software (NIH, Bethesda, MD). The femur length was recorded as appendicular length, and the spine length was recorded as axial length.

### Tissue Sample Length Measurements and Collection

After euthanasia, the femur and lumbar L5 vertebrae were fixed in 4% paraformaldehyde for at least 24 h. Ten percent ethylenediaminetetraacetic acid (EDTA) was used for decalcification of these bone tissues for at least 3 weeks. After decalcification, the femur and spine were embedded in paraffin for future histological analysis.

### Quantitative Histology of Axial and Appendicular Growth Plate Using H&E Staining

H&E staining and the quantitative analysis of GP were performed according to previous studies (23). Briefly, 4-μm-thick two nonconsecutive sections were made from paraffin of femur and lumbar (L5 vertebrae) tissues of each mouse and were then deparaffinized with xylene, followed by staining with H&E according to the manual protocol. The quantitative analysis was carried out by measuring the GP under the ×10 magnification using Nikon Eclipse microscope (Nikon, Japan). The height of GP was recorded by the mean values of 20 measurements per section. Furthermore, the height of the proliferative and hypertrophic zones was analyzed by 20 columns in each section and was recorded as an average. ImageJ (NIH, Bethesda, MD) was used to measure the height of the femur and lumbar GP height in a blind method.

### Immunohistochemistry

For immunohistochemistry (IHC) analysis, the traverse sections were baked for 50 min at 60°C, then rehydrated in xylene and graded ethanol, and finally washed with water, with 5 min in each step. Sections were incubated in 3% H_2_O_2_ in order to eliminate endogenous peroxidase activity; then phosphate-buffered saline (PBS) was used for washing. After retrieval and blocking, these sections incubated with rabbit antibody of anti-Collagen II (1:200, Affinity, USA, Catalog AF0135), anti-Collagen X (1:250, Affinity, USA, Catalog DF13214), anti-Aggrecan (1:200, Abcam, USA, Catalog ab216965), and anti-MMP13 (1:250, Affinity, USA, Catalog AF5355) at 4°C overnight. After being washed with PBS thrice, these slides were incubated with peroxidase-conjugated secondary antibody for 2 h at room temperature. Finally, these slides were incubated in ABC complex for 30 min. Staining was detected with 3,3′-diaminobenzidine (DAB) peroxide substrate solution for 3 min, followed by rinsing in distilled water. The sections were dehydrated by graded ethanol, cleared in xylene, and mounted with permount medium after counterstaining with Gill’s hematoxylin solution for 3 min. The results were analyzed and photographed by a digital microscope (Nikon, Japan).

### TUNEL Assay (Apoptosis Analysis)

The TUNEL assay was performed according to the manufacturer’s protocol (Roche, USA). Briefly, the apoptotic GP cells were identified by terminal deoxynucleotidyl transferase (TdT)-mediated deoxy UTP nick end labeling (TUNEL) IHC. The DAB method was used to detect the staining. The positive cells were counted within the GP and presented as a percentage of the total cells.

### Quantification of Immunohistochemistry Staining

The semiquantitative analysis method was used to evaluate the intensity of these immunoreactivity, measured by histological score (HSCORE) according to our previous study (23). The average results were addressed in a ×40 image. Briefly, the immunohistochemical localization was evaluated in a semiquantitative fashion focused on the intensity of staining area. The immunoreactive intensity of COL II, aggrecan, COL X, MMP13, ERα, and ERβ was assessed semiquantitatively according to categories of intensity: 0, no staining; 1, weak but detectable staining; 2, moderate or distinct staining; and 3, intense staining. In each field, the staining area was evaluated by summing the percentages of the area. The total score of each group was calculated as follows: HSCORE = Σi I × Pi (i represents the score of intensity, and Pi is the corresponding percentage of the areas).

### Femur Growth Plate Harvest and RNA Extraction

The distal femur or vertebrae were median incised using a surgical blade, and the GP can be seen under the field of a microscope. Micro-forcep was used to harvest the GP tissue. The harvested GP tissues were immediately placed in TRIzol reagent (Life Technologies). A homogenizer was used to make the homogenized GP. Then, the total RNA was extracted according to our previous studies (23, 25). NanoDrop spectrophotometer was used to quantify RNA, and 0. 5 μg was reverse transcribed into cDNA.

### Real-Time Quantitative PCR

Gene expression was evaluated by qPCR using a Roche LightCycler^®^ 480II (Roche Diagnostics). PCR amplification was performed with TB green premix Ex Taq (Takara) according to the manufacturer’s instructions. The forward and reverse primer sequences were as follows: ERα: 5′-CCTCCCGCCTTCTACAGGT-3′ and 5′-CACACGGCACAGTAGCGAG-3′; ERβ: 5′-CTGTGCCTCTTCTCACAAGGA-3′ and 5′-TGCTCCAAGGGTAGGATGGAC-3′; β-actin: 5′-GGCTGTATTCCCCTCCATCG-3′ and 5′-CCAGTTGGTAACAATGCCATGT-3′. The progress of qPCR contained two steps: first, an initial 30 s of heating to 95°C and then 20 cycles consisting of 5 s at 95°C followed by 20 s at 60°C. The ΔΔCt method was used to evaluate the transcript levels, and data were normalized for input based on β-actin and expressed relative to mice in the control group.

### Western Blotting Analysis

The distal femur or vertebrae were median incised using a surgical blade, and the GP can be seen under the field of a microscope. All GP tissues were lysed in radioimmunoprecipitation assay (RIPA) buffer (Beyotime, Jiangsu, China), and the protein concentration was analyzed using the bicinchoninic acid (BCA) kit (Beyotime, Jiangsu, China). The tissue lysates (35 μg) were separated by 10% sodium dodecyl sulfate–polyacrylamide gel electrophoresis (SDS-PAGE) using electrophoresis, and then the proteins were transferred to polyvinylidene difluoride (PVDF) membrane. After being blocked with 5% non-fat milk for 1 h, the membranes were incubated with primary anti-ERα (1:1,000, affinity, USA, Catalog No. AF6058), anti-ERβ (1:1,000, affinity, USA, Catalog No. AF6469), and GAPDH (1:5,000, ProteinTech, China, Catalog No. 10494-1-AP) at 4°C overnight. After being washed thrice with PBS, the membranes were incubated with an appropriate secondary antibody. Finally, the membranes were determined by Bio-Rad ChemiDoc (Bio-Rad Laboratories, Inc., USA) and were quantified by ImageJ software.

### Statistical Analysis

All the data were presented as mean ± SD. The unpaired t-test was used to analyze the differences in parameters between WT and ob/ob mice, the differences between control group and treated groups were analyzed by one-way ANOVA methods, and *post-hoc* analysis was carried out with Bonferroni’s test. Spearman’s correlation analysis was performed to correlate the HSCORE of ERα and limb/spine length. A *p*-value <0.05 was considered as statistically significant. Statistical analysis was performed using the SPSS (IBM, 22.0, USA) software.

## Results

**Figure 1:**
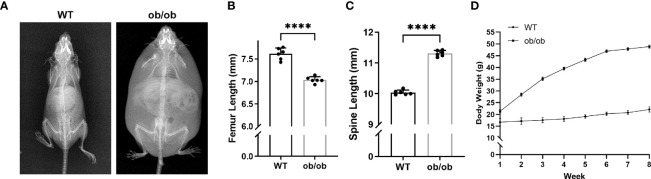
Comparison of body length between wild-type (WT) mice and ob/ob mice at the end of 8 weeks. **(A)** X-ray plain film of WT and ob/ob mice. **(B)** Femur length. **(C)** Spine length. **(D)** Body weight. Values are means ± SD. n = 6, ****p < 0.0001.

**Figure 2:**
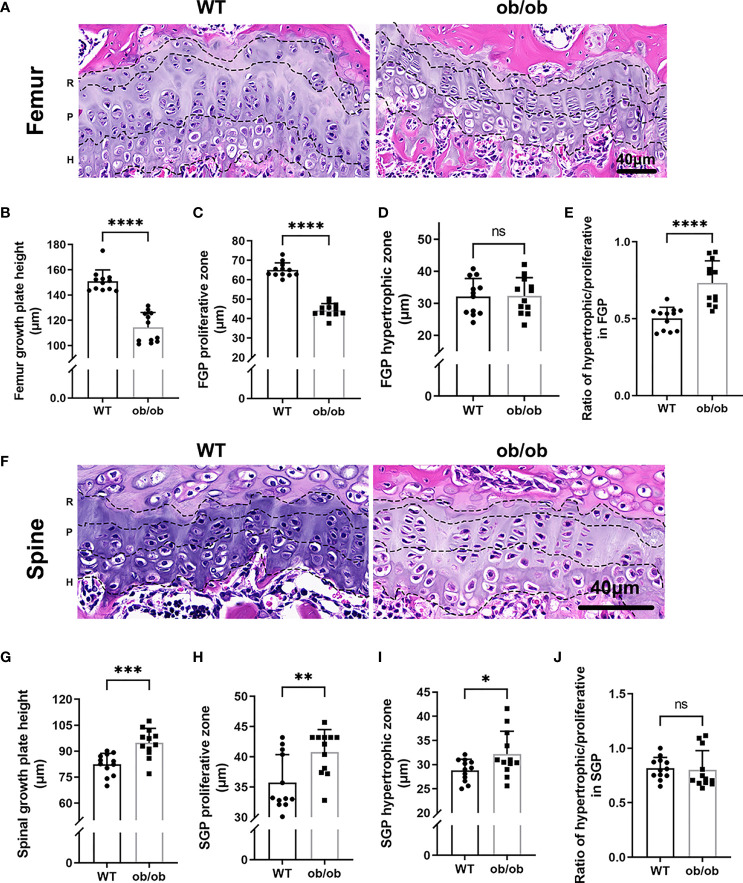
Comparison of femur and vertebrae growth plate length, proliferative and hypertrophic zone between wild type mice and ob/ob mice at the end of 8 weeks using HE staining method. R, resting zone; P, proliferative zone and H, hypertrophic zone. **(A)** Femur growth plate HE staining; **(B)** The height of femur GP; **(C)** The height of proliferative zone in femur GP; **(D)** The height of hypertrophic zone in femur GP; **(E)** Ratios of proliferative/hypertrophic zones in femur GP; **(F)** Spinal growth plate (SGP) HE staining; **(G)** The height of SGP; **(H)** The height of proliferative zone in SGP. **(I)** The height of hypertrophic zone in SGP; **(J)** Ratios of proliferative/hypertrophic zones in Spinal GP. Scale bar: 40μm; n=12 each group. Values are means±SD, *P < 0.05, **P < 0.01, ***P < 0.001 and ****P < 0.0001, ns denoted not significant.

At the end of mice’s puberty, X-ray was performed to collect images of the femur and lumbar spine. As shown in **Figure 1**, the femur of ob/ob mice was shorter than that of WT mice, and the lumbar length of ob/ob mice was longer than that of WT mice, which suggested that leptin deficiency mice had the contrasting phenotypes between femoral and lumbar spinal growth compared with those of WT mice. H&E staining of femoral and vertebral sections was used to investigate the thickness of GP in WT mice and ob/ob mice. The body weight (**Figure 1D**) of ob/ob mice was higher than that of WT mice at the beginning of the experiment (4-week-old mice), which was in line with the phenotype of obese ob/ob mice. The femur GP height of ob/ob mice was thinner than that of WT mice (**Figures 2A, B**), while the spinal GP of ob/ob mice was thicker than that of WT mice (**Figures 2F, G**). Additional histological analysis for the proliferative zone and hypertrophic zone of femur GP showed that both zones were decreased in the ob/ob mice compared with WT mice (**Figure 2C**), while the ratio of hypertrophic/proliferative zone of femoral GP was higher in ob/ob mice than in WT mice (**Figures 2D, E**). Located at the spinal GP, the proliferative zone in ob/ob mice was thicker than that of WT mice. However, there was no difference in the hypertrophic/proliferative zone ratio between ob/ob and WT mice (**Figures 2G**–**J**).

**Figure 3:**
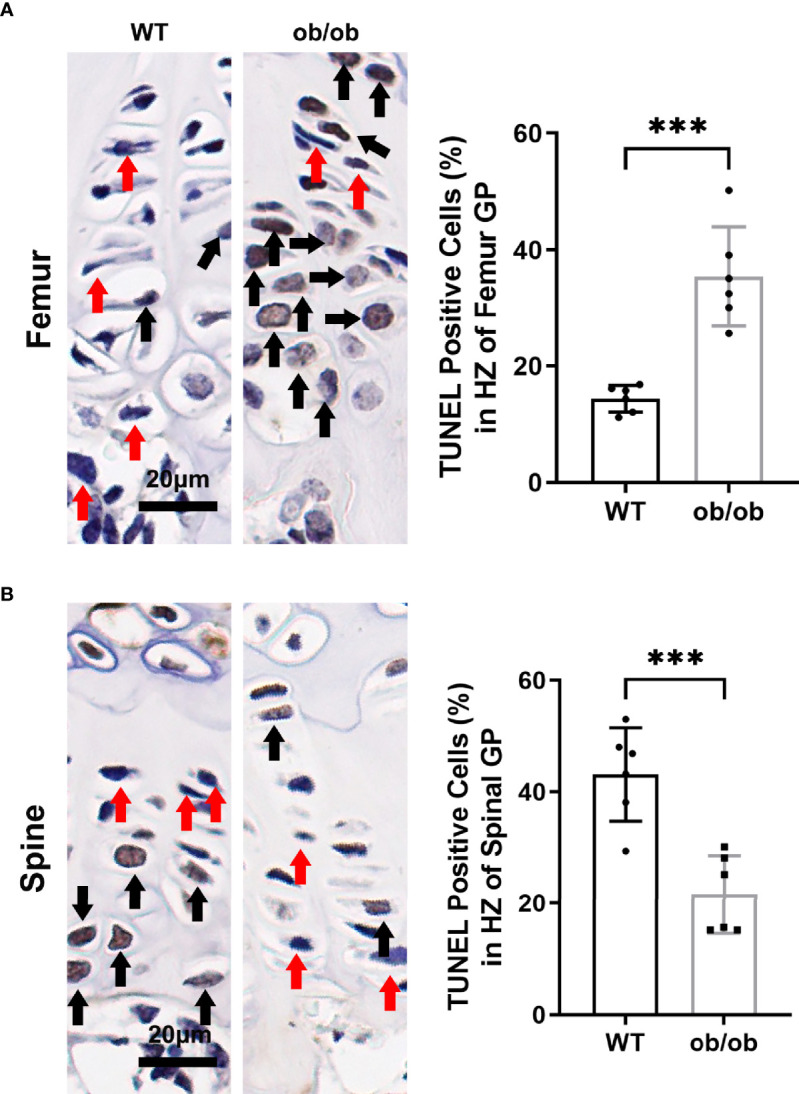
Comparison of femur **(A)** and spinal **(B)** growth plate apoptosis between wild type mice and ob/ob mice using TUNEL method, and percentages of positive cells were calculated. Black arrow determines the positive cell, and red arrow determines negative cell. Scale bar: 20μm; n=6 in each group, ***P < 0.001.

During the terminal differentiation procedure, GP hypertrophic chondrocytes undergo apoptosis with calcification of cartilaginous matrix followed by deposition of new bone. The positive number of TUNEL (+) chondrocytes at the hypertrophic zone were evaluated using a percentage of the total number of cells in the hypertrophic zone. At the end of our experiment (12-week-old WT mice), the mean percentage of apoptotic TUNEL-positive cells among the femur GP hypertrophic chondrocytes was 14.38 (**Figure 3A**). However, in 12-week-old ob/ob control mice, the percentage of apoptotic vertebral GP chondrocytes in the hypertrophic zone was greater (35.1%). The apoptosis rate in the vertebral GP chondrocytes of ob/ob mice was lower than that of WT mice (**Figure 3B**).

**Figure 4:**
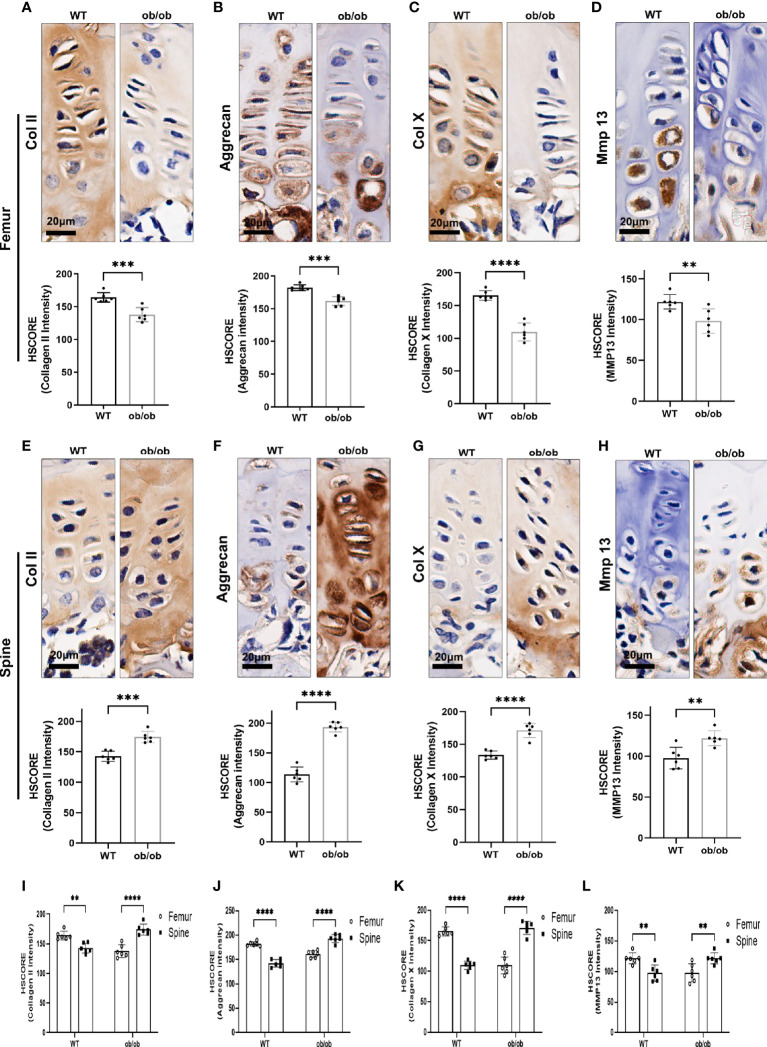
Comparison of femur and vertebral growth plate chondrocyte proliferation and hypertrophic markers between wild-type (WT) mice and ob/ob mice using immunohistochemistry (IHC) staining method, including collagen II **(A, E)**, aggrecan **(B, F)**, Col X **(C, G)**, and MMP13 **(D, H)**. Comparison of HSCORE of proliferation and hypertrophic markers between femur and vertebral growth plate in WT or ob/ob mice **(I–L)**. Scale bar: 20 μm; n = 6 in each group, **p < 0.01, ***p < 0.001, and ****p < 0.0001; ns denotes not significant.

**Figure 5:**
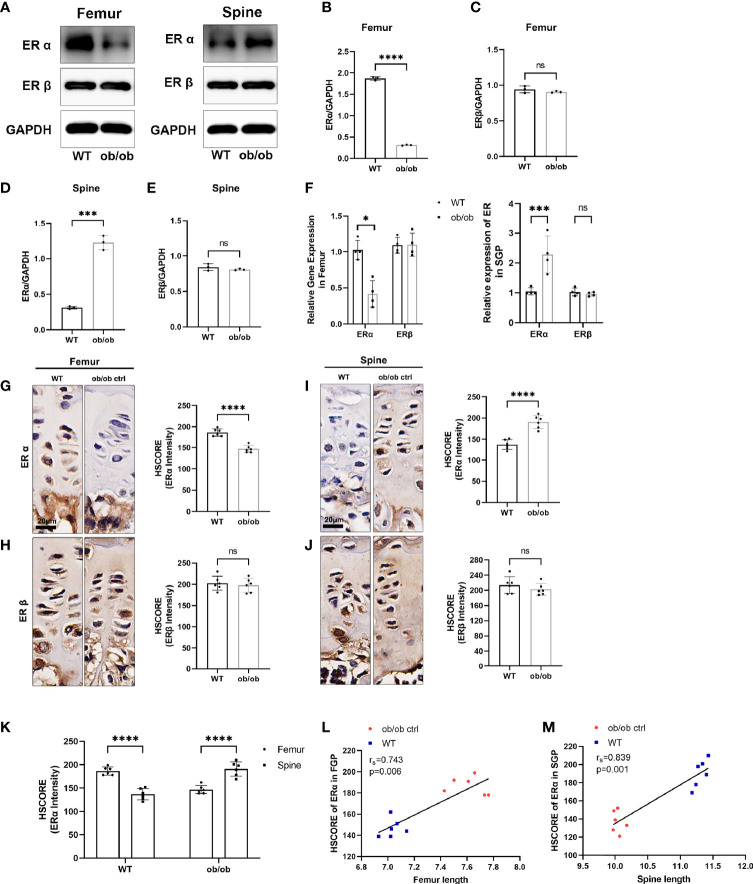
The expression level of estrogen receptors (ERs) in femur and spine. **(A)** Western blotting results of expression level of ERα and ERβ in femoral and spine growth plate (GP), n = 3. **(B–E)** Semiquantitative analysis of ER expression levels. **(F)** Quantitative real-time PCR results of ER genes in femoral and vertebral GP, n = 4. **(G–J)** Immunohistochemistry (IHC) results of expression level of ERα and ERβ in femoral and vertebral GP, n = 6. Scale bar: 20 μm. **(K)** Comparison of ERα HSCORE between femoral and spine GP (FGP, femoral growth plate; SGP, spine growth plate). **(L, M)** Results of Spearman’s analysis between ERα HSCORE and femur/spine length. *p < 0.05, ***p < 0.001 and ****p < 0.0001; ns denotes not significant.

The above results showed that ob/ob mice have opposite growth pattern between axial and appendicular growth compared with that of WT mice at the end of pubertal time. To determine the proliferation and hypertrophy fate of GP chondrocytes in the femur and spine, we performed IHC staining with proliferative marker (collagen II and aggrecan) and hypertrophic marker (collagen X and MMP13) on the femur and spinal GP (**Figure 4**). Collagen II and aggrecan were decreased in the femur GP of ob/ob mice compared with those of WT mice (**Figure 4**). Interestingly, we found that the expression of collagen II and aggrecan was higher in the spinal GP of ob/ob mice compared with the WT mice or the femur GP of ob/ob mice (**Figure 5**). Similarly, the expression of collagen X and MMP13 was also decreased in the femur GP of ob/ob mice, while the hypertrophic markers were highly expressed in the spinal GP of ob/ob mice. Overall, the reverse expression pattern of differentiation and hypertrophic markers demonstrated that leptin deficiency decreased the femur length *via* decreasing the expression of proliferative and hypertrophic marker genes and increasing the spinal length by enhancing these markers’ expression.

**Figure 6:**
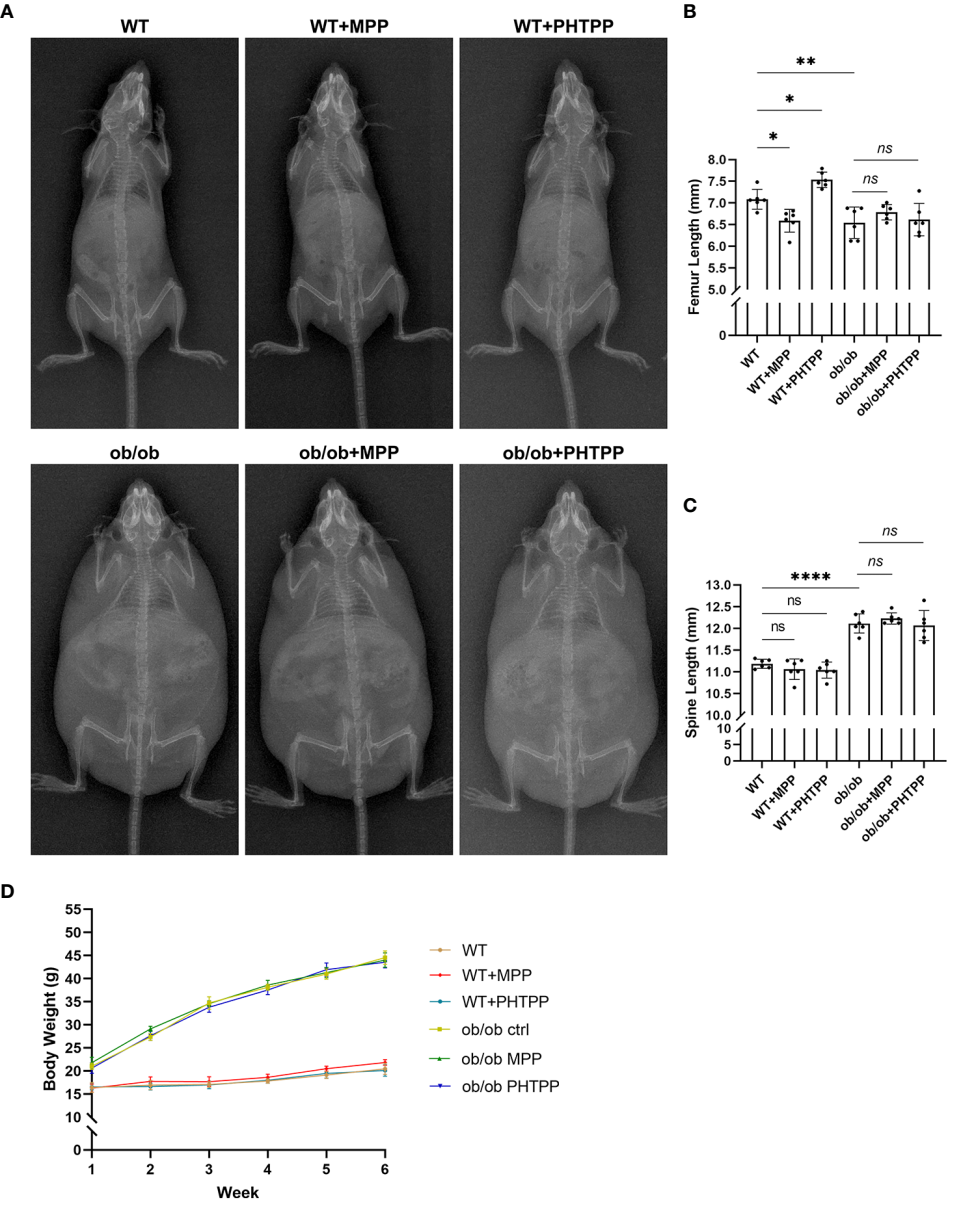
Leptin deficiency disturbs the regulatory effects of estrogen receptor-selective antagonists on the body length. **(A)** X-ray plain film of wild-type (WT) and ob/ob mice. **(B)** Femur length. **(C)** Spine length. **(D)** Body weight. Values are means ± SD. n = 6, *p < 0.05, **p < 0.01 and ****p < 0.0001; ns denotes not significant.

Previous studies have reported that ERs (α and β) play an important role in regulating the longitudinal bone growth. To further investigate mechanisms of contrasting growth phenotype of axial and appendicular growth, the Western blotting (WB), IHC, and qPCR methods were used to determine whether an opposite expression pattern of ERs existed in the femur and spinal GP (**Figure 6**). Interestingly, we found that the expression level of ERα was not only decreased in the femur GP of ob/ob mice. The spinal GP of ob/ob mice highly expressed the ERα compared with spinal GP of WT mice or the femur GP of ob/ob mice. In addition, the results of Spearman’s analysis showed that axial length and appendicular length were positively correlated with the expression level of ERα (**Figures 5L, M**). Collectively, the region-specific expression of ERα in the spinal and femur GP of ob/ob mice may play a role in the reverse growth pattern between axial and appendicular bone growth.

**Figure 7:**
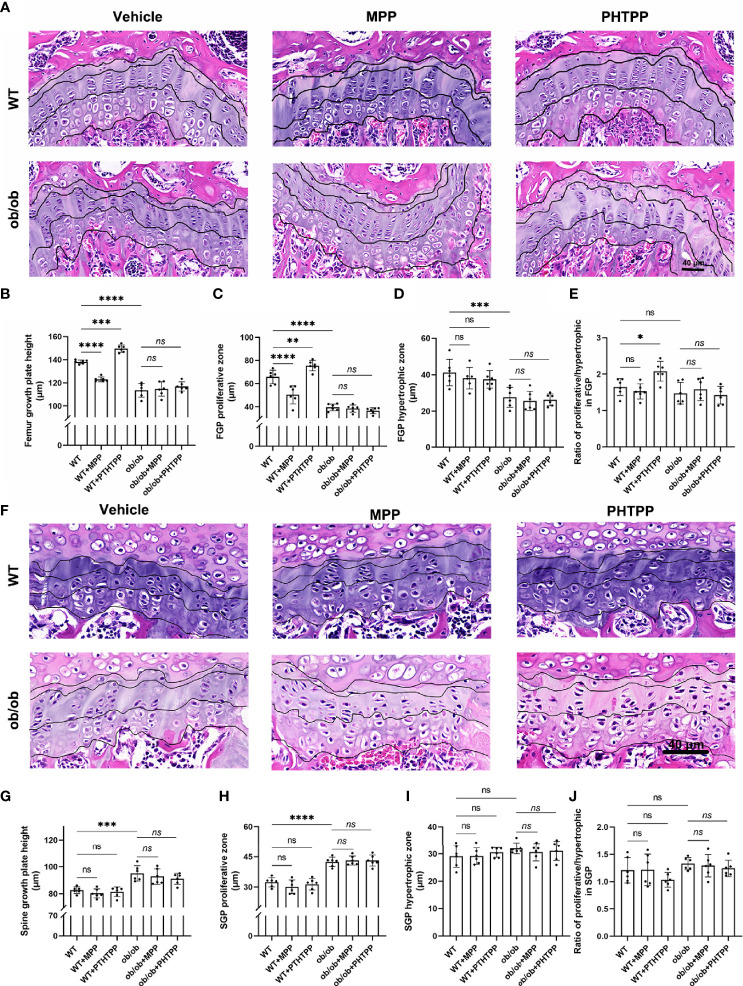
Leptin deficiency disturbs the regulatory effects of estrogen receptor-selective antagonists on the femur growth plate (GP) thickness and proliferative and hypertrophic zone using H&E staining method **(A–E)**. **(A)** Femur GP H&E staining. **(B)** The height of GP in the femur GP. **(C)** The height of proliferative zone in the femur GP. **(D)** The height of hypertrophic zone in femur GP. **(E)** Ratios of proliferative/hypertrophic zones in femur GP. Leptin deficiency disturbs the regulatory effects of estrogen receptor-selective antagonists on the vertebral GP length, and proliferative and hypertrophic zone using H&E staining method **(F–J)**. **(F)** Vertebral GP H&E staining. **(G)** The height of GP in the vertebral GP. **(H)** The height of proliferative zone in the vertebral GP. **(I)** The height of hypertrophic zone in vertebral GP. **(J)** Ratios of proliferative/hypertrophic zones in vertebral GP. Scale bar: 40 μm; n = 6 each group. Values are means ± SD, *p < 0.05, **p < 0.01, ***p < 0.001 and ****p < 0.0001; ns denotes not significant.

In order to determine the role of ERα in skeletal growth pattern of ob/ob mice, ERα and ERβ antagonists (MPP and PHTPP) were used. As shown in **Figure 7**, in the WT mice, blocking ERα led to a decreased femur length, and blocking ERβ resulted in an increased femur length compared with that in the non-treated WT mice. Furthermore, ER antagonists did not present regulative effects on the spinal length. The results of H&E staining, as shown in **Figure 7**, also showed that blocking ERα resulted in a thinner femur GP, and blocking ERβ increased the thickness of femur GP compared with that in non-treated WT mice, while ER antagonists did not influence the vertebral GP. These data were in line with our previous study (23). However, in ob/ob mice, any kind of ER antagonists did not present regulatory effects on the femur length and GP. Collectively, these data demonstrated that the absence of leptin might disturb the regulatory effects of ERs on the femur growth pattern. Precision mechanisms of region-specific ERα expression in femoral and vertebral GP in ob/ob mice still need to be explored in the future.

## Discussion

Although the contrasting phenotypes in bone of the limb and spine were observed in leptin deficiency mice, the reason and mechanisms remain unclear and need to be explored. The current study provides further evidence for the effects of leptin deficiency on the contrasting phenotypes on axial and appendicular bone growth, in which leptin deficiency results in region-specific expression of ERα at the femoral and vertebral GP in ob/ob female mice. According to the axial and appendicular bone length results, we showed that ob/ob mice have a shorter femur length and a longer spinal length than WT mice. IHC staining results also showed opposite results of proliferative and hypertrophic markers in femoral and spinal GP of ob/ob mice. Expression of ERs using IHC, WB, and qPCR methods demonstrated that femur GP has a lower level of ERα, and spinal GP has a higher level of ERα in ob/ob mice compared with those of WT mice. No differences were observed in the expression of ERβ of ob/ob mice compared with WT mice. Spearman’s analysis showed that body length was positively correlated with the expression level of ERα. Antagonists of ERs showed a different regulatory effect on femur length in WT mice, but blocking different ERs did not present regulatory effects on the skeletal longitudinal growth in ob/ob mice. Taken together, our data suggested that the contrasting phenotypes in bone growth of spine and limb induced by leptin deficiency may partially attribute to region-specific expression of ERα in the femoral and spinal GP. Leptin deficiency also blunted the regulatory effects of ER antagonists on the longitudinal growth in ob/ob mice. Still, the underlying mechanisms of the cross talk between leptin and ER signaling need to be studied in the future.

Puberty was the second growth peak in mammals. Except for the secondary sexual characteristics, body longitudinal growth was one of the important markers of the puberty (27). In addition to the regulation of metabolism and reproduction, leptin was reportedly important for the skeletal bone growth (12). Recently, leptin deficiency has been shown to lead to a shorter femoral length and a longer vertebrae length compared with WT mice using the 6-month-old and prepubertal female ob/ob mice (7, 28, 29). Supplementation of leptin has been shown to increase femoral length in ob/ob mice, but it could not achieve absolute re-established length as compared with WT mice (28, 30). In the present study, we confirmed that leptin deficiency induced a shorter femoral length and a longer vertebrae length in the post-pubertal (12-week old) ob/ob mice compared with WT mice, which suggested that the absence of leptin may be the primary factor for the contrasting growth pattern between limb and spine during the whole life of ob/ob female mice. Previous studies have shown that leptin receptor was expressed in cartilaginous skeletal growth centers, articular cartilage, and osteoblasts (31, 32). Our previous study also showed that leptin receptor was expressed in the femur and spinal GP (33). The *in situ* expression of leptin receptor at the GP supported that leptin directly affected the GP. GP thickness was an important histological marker for the longitudinal bone growth (34). Here, we focused on the GPs and found that the femoral GP was thinner than that of WT mice, and the spinal GP was thicker than that of WT mice. Leptin deficiency decreased the proliferative zone and hypertrophic zone height in the femoral GP compared with the WT mice, but the ratio of hypertrophic/proliferative zone was higher in ob/ob mice. The contrasting GP patterns of the femur and vertebrae were in line with the general femoral and spinal length observation. As we know, endochondral ossification was the major process that contributed to skeletal bone longitudinal growth (9). During this procedural transition from cartilage to bone, the potential abilities of chondrocyte proliferation and hypertrophy were important for GP thickness and were further responsible for longitudinal bone growth (35). Chondrocytes in different phases of GP secreted unique matrix proteins. Collagen II and aggrecan were used as the markers of GP cellular proliferation. Meanwhile, collagen X and MMP13 were the typical features of GP hypertrophy (36–38). The results of our study demonstrated that leptin deficiency decreased the expression of extracellular matrix marker proteins in femur GP as compared with those in the WT mice, which was in line with the results of Tuner and colleagues (28). Interestingly, the extracellular matrix makers were at a higher level in the spinal GP as compared with those in the spinal GP of WT mice and the femur GP of ob/ob mice. Collectively, the results of the extracellular matrix maker expression patterns in femoral and spinal GP support the contrasting phenotypes in bone growth of the limb and spine.

To explore the mechanism of this interesting phenomenon, we further focused on the expression of ERs in the femoral and spinal GP. Estrogen was a key hormone in puberty of female mammals, and it was also an important factor that may influence the process of skeletal bone line growth (36). ERs mediated cellular response of estrogen, and previous studies using different global ER knock-out mouse models have suggested that ERα and ERβ presented opposing effects on longitudinal bone growth in female mice (20, 22, 39). ERβ knock-out adult mice have increased skeletal length compared with WT female mice, while ERα knock-out mice presented decreased longitudinal bone growth (20, 22). A previous study has shown that the expression level of ERs (especially ERα) was high at the arcuate nucleus in ob/ob mice (40). Furthermore, in our previous studies (24, 25), there were cross-talk effects between leptin and ERs *in vitro*. However, no reports have been focused on the expression pattern of ERs located at the spinal and femoral GP in ob/ob mice *in vivo*. In the present study, our data first showed that the expression level of ERα was lower in the femoral GP of ob/ob mice compared with the WT mice, and the level of ERα was higher in the spinal GP of ob/ob mice compared with WT mice or the femoral GP of ob/ob mice. In addition, there were no significant changes in ERβ located at the femoral and spinal GP. ERα was considered as a growth promoter in the skeletal development (20, 39). These interesting findings of our study may partially explain the mechanisms of contrasting phenotypes in bone growth of limb and spine. Low expression of ERα in femoral GP resulted in short limbs, and high expression of ERα in spinal GP resulted in long vertebrae. The expression level of ERα was positively correlated with axial and appendicular bone length according to the results of Spearman’s analysis. Furthermore, ER antagonists were used to determine the role of ERα in regulating the longitudinal bone growth. In line with our previous study (23), the present data showed that ERα blocking directly resulted in decreasing appendicular longitudinal growth, whereas ERβ blocking led to increasing appendicular longitudinal growth in the WT mice. However, selective ER antagonists did not present regulative effects on the longitudinal growth of the ob/ob mice. Taken together, our results show that leptin deficiency directly induced the different expression patterns of ERα at femoral and vertebral GP and low serum estradiol level in ob/ob mice during puberty, and the regulative effects of ER antagonists were disturbed by the absence of leptin. Given the complex effects of leptin deficiency in regulating the bone growth, more specific mechanisms are needed to be explored.

In conclusion, we found that leptin deficiency presented contrasting phenotypes in axial and appendicular growth of ob/ob mice at the end of puberty, with reduced level of proliferative and hypertrophic markers in femoral GP and an increased level of these markers in spinal GP. Furthermore, we first found a significant change of expression of ERα located at femoral and spinal GP (lower in femoral GP and higher in spinal GP as compared with that in WT mice). These findings suggested that region-specific expression of ERα might be associated with the formation of contrasting phenotypes of axial and appendicular bone growth in the ob/ob mice. However, further studies are needed to clarify the relation between ERs and leptin in regulating endochondral ossification.

## Data Availability Statement

The original contributions presented in the study are included in the article/supplementary material. Further inquiries can be directed to the corresponding authors.

## Ethics Statement

The animal study was reviewed and approved by the animal experiment ethical committee of Peking University People’s Hospital.

## Author Contributions

X-FL and H-YL designed the study. L-YJ and CG performed all experiments. L-YJ and SX analyzed data. L-YJ prepared the figures. L-YJ and X-FL wrote the manuscript. All authors contributed to the article and approved the submitted version.

## Funding

This work was supported by grants from the National Natural Science Foundation of China (No. 81772292) and the Procurement of Government of National Health Commission of China (No. 2127000218).

## Conflict of Interest

The authors declare that the research was conducted in the absence of any commercial or financial relationships that could be construed as a potential conflict of interest.

## Publisher’s Note

All claims expressed in this article are solely those of the authors and do not necessarily represent those of their affiliated organizations, or those of the publisher, the editors and the reviewers. Any product that may be evaluated in this article, or claim that may be made by its manufacturer, is not guaranteed or endorsed by the publisher.
